# Beam Angle Optimization for Double-Scattering Proton Delivery Technique Using an Eclipse Application Programming Interface and Convolutional Neural Network

**DOI:** 10.3389/fonc.2021.707464

**Published:** 2021-09-14

**Authors:** Wonjoong Cheon, Sang Hee Ahn, Seonghoon Jeong, Se Byeong Lee, Dongho Shin, Young Kyung Lim, Jong Hwi Jeong, Sang Hee Youn, Sung Uk Lee, Sung Ho Moon, Tae Hyun Kim, Haksoo Kim

**Affiliations:** Proton Therapy Center, National Cancer Center, Goyang-si, South Korea

**Keywords:** deep-learning, convolutional neural network, beam angle optimization, proton therapy, double-scattering technique

## Abstract

To automatically identify optimal beam angles for proton therapy configured with the double-scattering delivery technique, a beam angle optimization method based on a convolutional neural network (BAODS-Net) is proposed. Fifty liver plans were used for training in BAODS-Net. To generate a sequence of input data, 25 rays on the eye view of the beam were determined per angle. Each ray collects nine features, including the normalized Hounsfield unit and the position information of eight structures per 2° of gantry angle. The outputs are a set of beam angle ranking scores (*S*_beam_) ranging from 0° to 359°, with a step size of 1°. Based on these input and output designs, BAODS-Net consists of eight convolution layers and four fully connected layers. To evaluate the plan qualities of deep-learning, equi-spaced, and clinical plans, we compared the performances of three types of loss functions and performed *K*-fold cross-validation (*K* = 5). For statistical analysis, the volumes V_27Gy_ and V_30Gy_ as well as the mean, minimum, and maximum doses were calculated for organs-at-risk by using a paired-samples *t*-test. As a result, smooth-L1 loss showed the best optimization performance. At the end of the training procedure, the mean squared errors between the reference and predicted *S*_beam_ were 0.031, 0.011, and 0.004 for L1, L2, and smooth-L1 loss, respectively. In terms of the plan quality, statistically, Plan_BAO_ has no significant difference from Plan_Clinic_ (*P* >.05). In our test, a deep-learning based beam angle optimization method for proton double-scattering treatments was developed and verified. Using Eclipse API and BAODS-Net, a plan with clinically acceptable quality was created within 5 min.

## Introduction

Interest in beam angle optimization (BAO) research has been on the rise recently again. When intensity-modulated radiotherapy (IMRT) emerged as a novel treatment method, BAO research was being actively undertaken. IMRT could achieve high dose conformity while minimizing undesirable dose to organs-at-risk (OARs). However, the conventional BAO process for the IMRT plan is based on trial-and-error searching by a planner; the optimal beam angle is affected by the experience and understanding of the treatment planning system (TPS) of the planner (1). Thus, various studies for BAO have been conducted to reduce the workload of treatment planning and decrease the planning time. These BAO studies incorporated techniques such as simulated annealing (2–9), geometric information scoring (10–19), gradient descent (20–25), genetic algorithms (26–29), and neural networks (30–34). However, the advent of volumetric-modulated arc therapy and the templatization of the radiation treatment plan, including dose prescription and gantry angles, have reduced interest in BAO research for X-ray therapy.

Recently, with increasing interest in proton and heavy ion therapy, which rely on the characteristics of a Bragg peak and a relatively higher radiation biological effect than X-ray therapy, several recent studies on intensity-modulated ion therapy (35) in BAO research have been published (1, 36–39).

The present study was inspired by two previous studies on BAO. In 1999, Hosseini-Ashrafi et al. conducted a study on the BAO of X-ray therapy in which they used an artificial neural network (ANN) (30). In that study, the radiation treatment plans were divided into several templates, and the ANN classified the test data according to the template. The ANN consisted of three layers of a multi-layer perceptron. The input contained 12 pre-calculated features, which were the body contour outline, treatment volume, sensitive organs, and border of tissue inhomogeneity for each case. The output contained three types of binary data for eight classification tasks. The ANN was validated using the leave-one-out method, which showed the feasibility of applying ANNs to the BAO problem.

In 2002, Pugachev et al. published a research paper on BAO for IMRT (4). They proposed beam’s eye view dosimetrics (BEVD) to overcome low computation speed, which is a disadvantage of the simulated algorithm. The BEVD score was calculated by using the geometric and dosimetric information of the patient. This score was used for ranking information and as a prescreening tool to optimize the beam orientation by using a simulated annealing-based BAO algorithm. The treatment plans generated with the guidance of the BEVD score were compared with those created with five equiangular-spaced beams. They validated the feasibility of the BEVD score for the BAO problem. The BEVD guidance indicated that the computational efficiency increased by a factor of ~10.

In the current study, we developed a deep-learning based BAO method for the three-ports proton double-scattering (DS) technique using the geometric information of the patient computed tomography (CT) anatomy and Hounsfield unit (HU) data as well as a convolutional neural network (CNN). In particular, the DS technique was used for large-field proton therapy. The proposed method requires only geometric information without a fluence optimization process. The geometric information is automatically extracted using an application programming interface (API) of a TPS (Eclipse, Varian, Palo Alto, CA, USA). A set of beam angle ranking scores (*S*_beam_) for all angles is predicted using the deep-learning model. The quality of the treatment plans created with the predicted *S*_beam_ guidance was statistically compared for equi-spaced and clinical plans. The evaluation was performed using the dose–volume histogram (DVH) parameters.

## Materials and Methods

### Patient Database

Patient data from 50 liver cases, consisting of average intensity projection (AIP) CT images calculated from the four-dimensional (4D) CT images of 40–60% phases, a digital image communication in medicine-radiation therapy (DICOM-RT) structure file, and a DICOM-RT plan file, were used in this study. The patients were originally treated using the proton DS delivery technique configured with three DS fields at the National Cancer Centre in the Republic of Korea (40).

To automatically access information of interesting structures in the TPS, an in-house software was developed using the Eclipse script API. The eight structures of interest included the body, total liver volume (TLV), primary gross tumor volume (PGTV), duodenum, stomach, esophagus, heart, and spleen. The body contour included areas such as immobilizers that should be considered for dose calculation.

### Geometric Information and a Set of Beam Angle Ranking Scores for Beam Angle Optimization: Input and Output of the Deep-Learning Model

To train a beam angle optimization network (BAODS-Net), geometric information extracted from the AIP CT images and DICOM-RT structures (an input of BAODS-Net) and *S*_beam_ generated from RT-plan (an output of BAODS-Net) were used.

**Figure 1:**
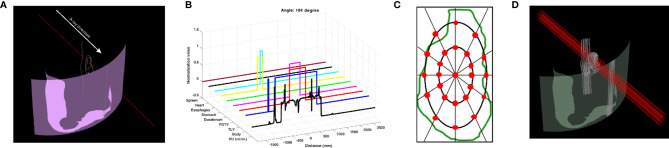
Data collector of patient CT anatomy and Hounsfield unit (HU) data used as input to BAODS-Net. **(A)** Data collector in 3D. **(B)** Extracted patient anatomy and HU data. **(C)** Twenty-five data collectors in the cross-section of the primary gross tumor volume at the isocenter plane. **(D)** Final data collectors consisting of 25 rays per angle.

The geometric information was collected by the ray tracing method (4). A ray, which is a collector, was determined to penetrate from the body contour to the isocenter, and the path of the ray was tracked in a 3D treatment room coordinate system. The ray collected geometric information by dividing the length of 1,000 mm into 4,000 bins (**Figure 1A**). The collected data included the following: specifically HU from the AIP CT images as a double data type and the anatomy position information of interest of eight structures as a binary data type (**Figure 1B**). Thus, the shape of geometric information extracted from a collector was 9 × 4,000. Geometric information collected by the penetrating ray is useful data to evaluate the best DS field that considers range uncertainties and OAR positions. In this regard, additional 24 parallel rays penetrating the target volume were created. In detail, the positions of the 24 rays were automatically determined at an isometric angle on two ellipses with different radii placed on the PGTV cross-section in the isocenter plane observed from the beam's eye view (**Figure 1C**). Thus, at a specific gantry angle, 25 data collectors extracted nine geometric information points (**Figure 1D**). In the same manner, the patient CT anatomy and HU data for all directions were collected at angles from 0° to 358° in steps of 2°. Finally, the shape of the geometric information extracted by the 25 data collectors on coplanar was 40,500 × 4,000. The geometric information was reshaped to input 4D tensor (batch, channel, height, and width). For pre-processing, only the HU values were normalized by using Z-score normalization per patient. The Z-score normalization is a standardization method for the fast convergence of deep-learning models. For the case of the anatomy position information of eight structures, normalization was not performed.

**Figure 2:**
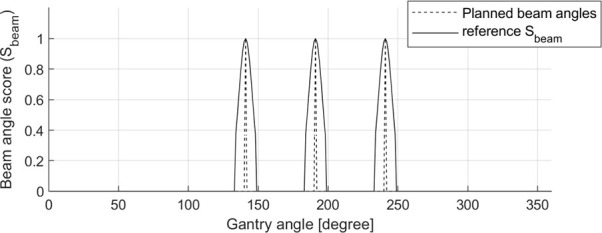
Sample of planned beam angles (140°, 190°, and 240°) and a reference set of beam angle ranking scores (*S*_beam_) for BAODS-Net.

A *S*_beam_ ground-truth for each patient was generated using the gantry angle information in the DICOM-RT plan files with steps of 1°. The *S*_beam_ comprised one-dimensional (1D) data continuously ranging from 0.0 to 1.0. The gantry angles used in the clinic were assigned a value of 1.0; otherwise, a value of 0.0 was assigned. Then, to induce effective optimization of BAODS-Net, a normalized Gaussian filter was applied. Finally, the size of *S*_beam_ was 360, and the shape of *S*_beam_ was reshaped to a batch-considered shape. An example of a reference *S*_beam_ is shown in **Figure 2**.

K-fold cross-validation (CV) could provide a better indication of how well the BAODS-Net was universalized to unobserved data. We performed a patient-wise K-fold CV method (*K* = 5), and all datasets were divided into five disjointed and identically sized subsets (41).

### Double-Scattering Beam Angle Optimization Network

**Figure 3:**
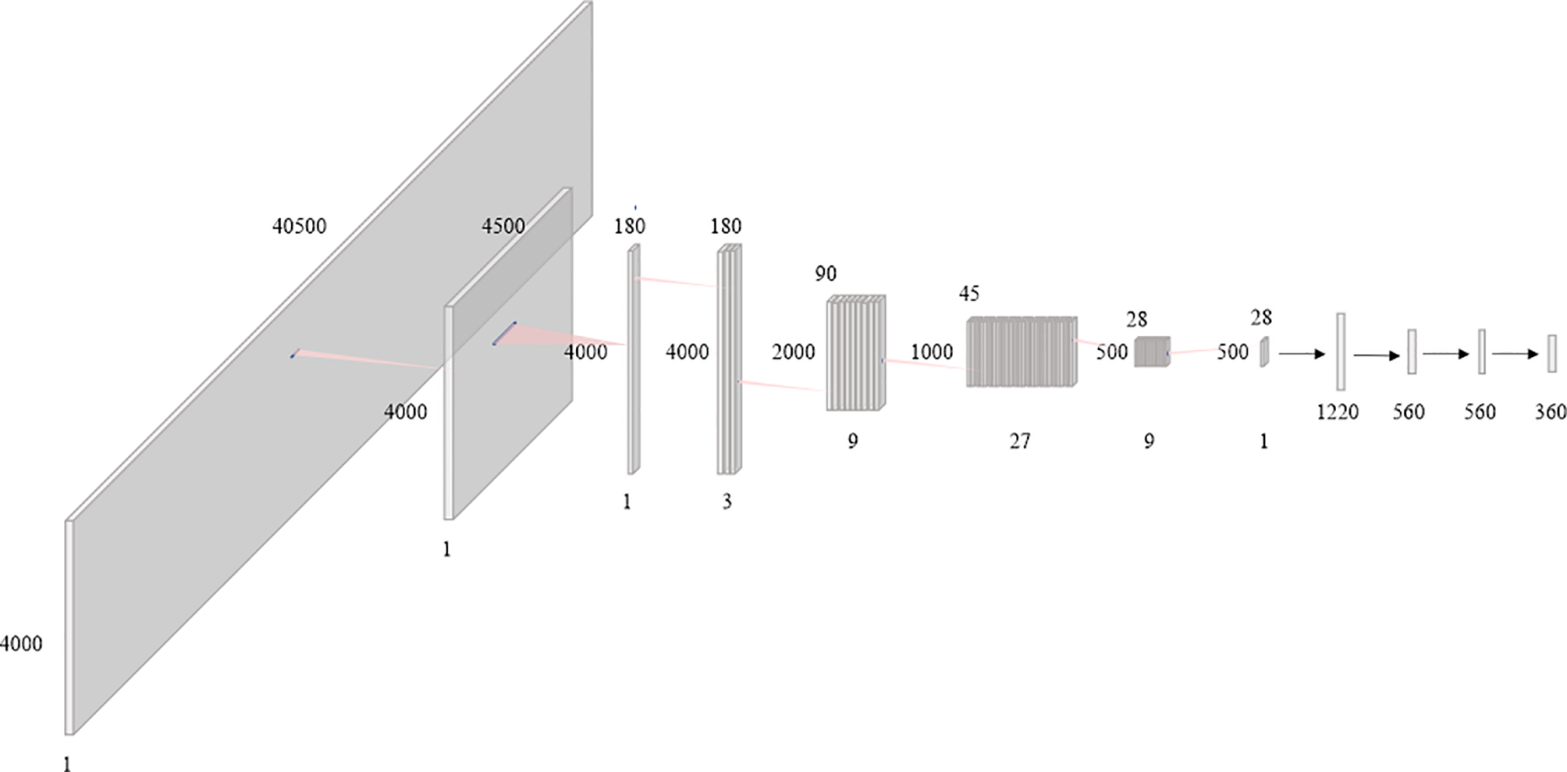
Diagram of the proposed beam angle optimization for proton double-scattering network (BAODS-Net).

In this paper, the BAODS-Net based on a CNN is proposed as shown in **Figure 3**. It consists of two main stages: a feature extractor and a predictor. The feature extractor was configured with eight convolution layers to extract distinguishable features of the geometric information by applying a convolution layer with various strides. The first convolution layer was designed with dimensions of 1 × 9 for the width and height, respectively. The convolution layer was operated with a stride size of nine and with the same padding option. This is because the nine geometric features extracted by a ray are intended to be integrated into a weighted geometric feature. The second convolution layer was designed with dimensions of 1 × 25 for the width and height, respectively. A stride size of 25 was used to integrate each weighted geometric feature extracted from the 25 rays into one weighted ray representing a specific angle. The remaining part of the feature extractor was designed with six convolution layers (width: 3, height: 3, stride: 1) and max-pooling layers. The output of the feature extractor was flattened and then passed to the predictor. The predictor was designed with four fully connected (FC) layers to continuously predict *S*_beam_ from 0° to 359°. Although the FC layer is computationally expensive, it can effectively predict *S*_beam_ with a non-linear activation function because the FC layer has a structure agnostic property.

Batch normalization was applied for fast convergence in the optimization process and to ensure the robustness of the performance (42). Randomly initialized biases were added for all layers. Additionally, the activation function was the leaky rectified linear unit (43), which was used to maintain the contribution of negative data, and the adaptive momentum estimation optimizer was employed (44). The BAODS-Net trained for 5,000 epochs with a learning rate of 0.001 and weight decay of 0.0002, and to compute the running average of the gradient, β_1_ was 0.9 and β_2_ was 0.999. The output shape and parameters of the layers composing the BAODS-Net are summarized in **Table 1**.

**Table 1:** The architecture of the proposed BAODS-Net.

	Layer	Output Shape (B, C, W, H)	Kerner Size
Feature extractor	Conv2d	(None, 1, 4500, 4000)	(1, 1, 9, 1)
	BatchNorm2d	(None, 1, 4500, 4000)	2
	Conv2d	(None, 1, 180, 4000)	(1, 1, 25, 1)
	BatchNorm2d	(None, 1, 180, 4000)	2
	Conv2d	(None, 3, 178, 3998)	(3, 1, 3, 3)
	BatchNorm2d	(None, 3, 178, 3998)	6
	MaxPool2d	(None, 3, 89, 1999)	0
	Conv2d	(None, 9, 87, 1997)	(9, 3, 3, 3)
	BatchNorm2d	(None, 9, 87, 1997)	18
	MaxPool2d	(None, 9, 43, 998)	0
	Conv2d	(None, 27, 41, 996)	(27, 9, 3, 3)
	BatchNorm2d	(None, 27, 41, 996)	54
	MaxPool2d	(None, 27, 20, 498)	0
	Conv2d	(None, 9, 18, 496)	(9, 27, 3, 3)
	BatchNorm2d	(None, 9, 18, 496)	18
	MaxPool2d	(None, 9, 9, 248)	0
	Conv2d	(None, 3, 7, 246)	(3, 9, 3, 3)
	BatchNorm2d	(None, 3, 7, 246)	6
	Conv2d	(None, 1, 5, 244)	(1, 3, 3, 3)
	BatchNorm2d	(None, 1, 5, 244)	2
Predictor	Linear	(None, 560)	(560, 1220)
	BatchNorm1d	(None, 560)	1,120
	Linear	(None, 560)	(560, 560)
	BatchNorm1d	(None, 560)	1,120
	Linear	(None, 560)	(560, 560)
	BatchNorm1d	(None, 560)	1,120
	Linear	(None, 360)	(360, 560)

B, batch size; C, channel; W, width; H, height; Conv2d, two-dimensional (2D) convolution layer; BatchNorm2d, 2D batch normalization layer; MaxPool2d, 2D Max pooling layer; Linear, fully connected layer; BatchNorm1d, one-dimensional batch normalization layer.

### Training and Validation of BAODS-Net

In the training process, BAODS-Net was optimized to predict *S*_beam_ by using the training data. To find the training loss function that could achieve the best BAODS-Net performance, we compared the performances of the three types of loss functions: L1 loss (Eq. 1), L2 loss (Eq. 2), and smooth-L1 loss (Eq. 3). The prediction accuracy for *S*_beam_ was calculated by using mean squared error (MSE) between the reference and predicted *S*_beam_.


(1) $$\text{L}1\;\text{loss}(x,\text{y})=\frac{1}{n}\sum_{\text{i}}{\text{Z}}_{i},{\text{Z}}_{i}=\left|{\text{x}}_{i}-{\text{y}}_{i}\right|,$$

(2) $$\text{L}2\;\text{loss}(\text{x},\text{y})=\frac{1}{\text{n}}\sum_{\text{i}}{\text{Z}}_{\text{i}},{\text{Z}}_{i}={\left({\text{x}}_{\text{i}}-{\text{y}}_{\text{i}}\right)}^{2},$$

(3) $$\text{Smooth L}1\;\text{loss}(\text{x},\text{y})=\frac{1}{\text{n}}\sum_{i}Z_{i},Z_{i}=\{\begin{array}{l} 0.5\frac{{\left({\text{x}}_{i}-{\text{y}}_{i}\right)}^{2}}{\beta },i\text{f}\left|{\text{x}}_{i}-{\text{y}}_{i}\right|<\beta \\ \left|{\text{x}}_{i}-{\text{y}}_{i}\right|-0.5\beta ,\;\text{otherwise} \end{array}$$

where (*x*, *y*) is the reference *S*_beam_ and predicted *S*_beam_, respectively, and *n* is the number of samples. The hyper-parameter beta (*β*) in Eq. 3 was a value for applying additional weight to the loss. *β* was empirically determined to be 0.5.

In this experiment, the data of the first fold were used. Specifically, the smooth-L1 loss could be interpreted as a combination of L1 and L2 losses.

To improve the BAODS-Net performance and reduce its generalization error, a 1D augmentation technique, which is a 1D data translation ranging from −2 to 2°, was applied to the reference *S*_beam_. The augmentation data were randomly generated for each epoch. Through the K-fold CV principle, we independently conducted five different runs for five separate CV datasets to evaluate the BAODS-Net performance.

### Plan Creation With BAODS-Net

The procedure for creating a three-ports proton DS plan with the guidance of BAODS-Net (Plan_BAO_) was as follows: (i) the patient CT anatomy and HU data were automatically extracted by using an in-house software based on Eclipse API, (ii) the patient CT anatomy and HU data were fed into the BAODS-Net, and then the BAODS-Net output (*S*_beam_) was predicted; (iii) the specific angles in *S*_beam_ were selected according to a selection rule. The rule preferentially selected the three gantry angles corresponding to the highest score. However, if the interval was less than 30°, the next priority angle was selected; (iv) the collimator and compensator were designed using the default TPS option without manual modification for objective evaluation of BAODS-Net performance, and (v) the field weight was set to one for all fields.

### Plan Comparison of BAODS-Net, Equi-Spaced Angle, and Clinical Plan

To validate the liver treatment plan quality for 50 patients, results were obtained by combining the results of the five folds. The DVH parameters were analyzed for Plan_BAO_, the equi-spaced plan [gantry angles were fixed at 0°, 120°, 240° (Plan_Equi_)], and the clinical plan (Plan_Clinic_). Although the equi-spaced plan is rarely applied in clinics for proton beam by a planner, we added to the equi-spaced plan for comparative study (4). The Plan_Clinic_s were created by a qualified planner with 5 years of clinical experience. The evaluation metric is defined below, and the conformity index (CI) was calculated for PGTV (Eq. 4).


(4) $$\text{Conformity index}=\frac{\text{TV}\times \text{PIV}}{\text{T}{\text{V}}_{\text{PIV}}^{2}},$$

where TV is the target volume and PIV is the prescribed isodose volume. The closer PIV is to TV, the closer the CI is to 1. The volumes V_27Gy_ and V_30Gy_ for TLV as well as the mean, minimum, and maximum doses for OARs were calculated. V_xGy_ represents the volume percentage of the whole organ receiving a dose ≥xGy. For statistical analysis, these results were compared with the paired-samples *t*-test. All statistical analyses were implemented using SAS 9.4 software (SAS Institute Inc., Cary, NC, USA), and the statistically significant level was set at *P* = .05.

## Result

### Performance Comparison of L1, L2, and Smooth-L1 Loss Functions

To determine the optimal loss function, BAODS-Net was trained using L1, L2, and smooth-L1 loss. The training time was approximately 120 h by using the training data of the first fold, which corresponded to 5,000 epochs when using an NVIDIA Quadro GV100 graphics processing unit (GPU) (NVIDIA, Santa Clara, CA, USA). The seed number was fixed in the training procedure. The MSE between the predicted and reference *S*_beam_ was evaluated when L1, L2, and smooth-L1 loss were used for each model training procedure. At 5,000 epochs, the MSEs were 0.031, 0.011, and 0.004 for L1, L2, and smooth-L1 loss, respectively. As a result, the smooth-L1 loss was adopted as a metric for the training loss.

### Plan Comparison of BAOBS-Net, Equi-Spaced Angle, and Planner

To evaluate the cases of 50 patients as test data, BAODS-Net was trained and evaluated with five different folds, and the training losses in the five different runs were recorded. At the 5,000th epoch, the mean and standard deviation of MSEs between the predicted and reference *S*_beam_ for the five folds were 0.0037 and 0.0006, respectively.

**Figure 4:**
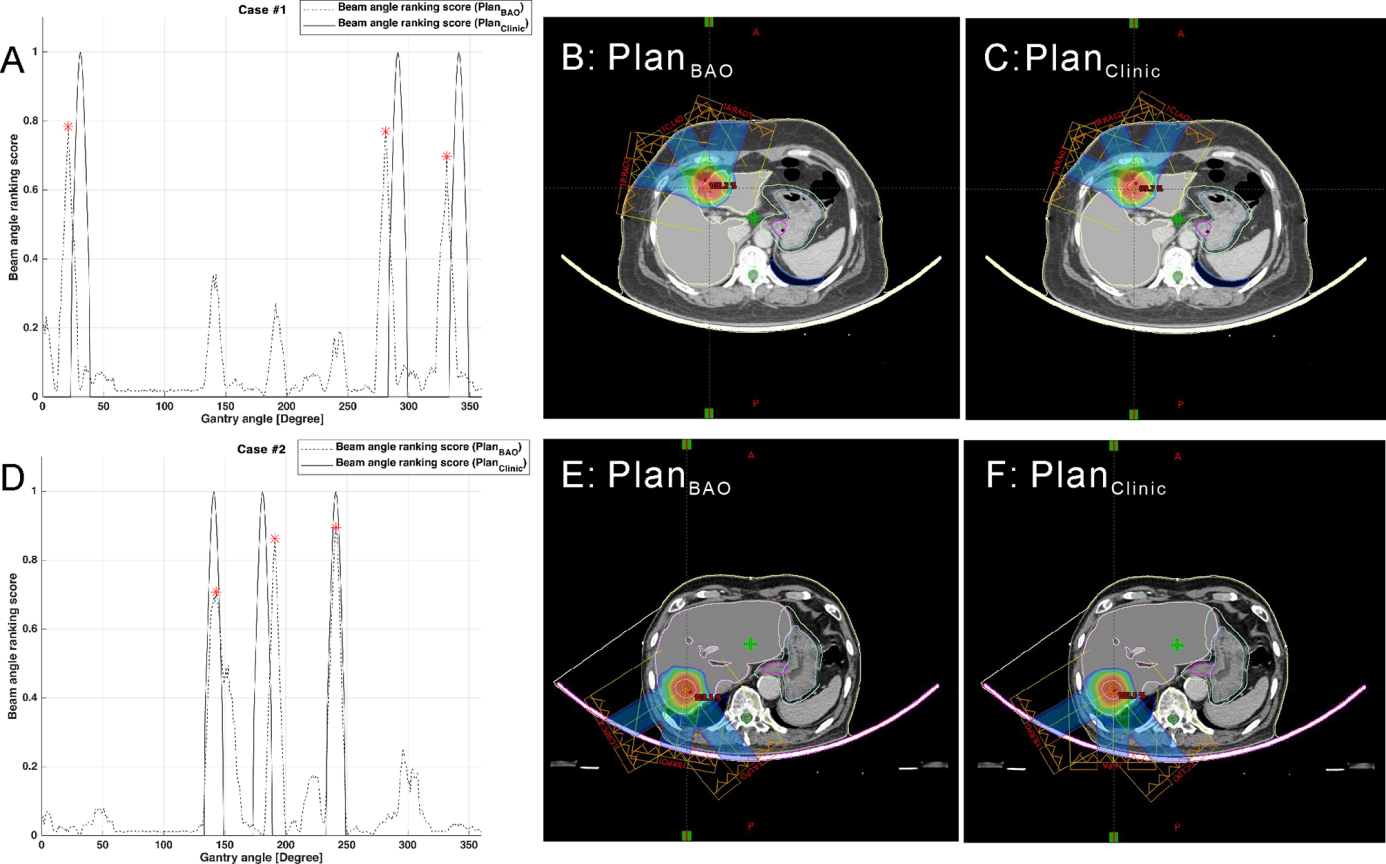
Two test cases of reference and predicted *S*_beam_ and 2D dose distribution at the isocenter plane: case 1 **(A–C)** and case 2 **(D–F)**.

The plans created by guidance with BAODS-Net, equi-spaced angle, and the planner method were compared using the DVH parameters. In **Figure 4**, the paired two test cases of Plan_BAO_ and Plan_Clinic_ are visually analyzed, including the reference, predicted *S*_beam_, and 2D dose distribution at the isocenter plane. The star markers in **Figures 4A, D** are the gantry angles finally selected for Plan_BAO_.

Data from a total of 37 out of 50 patients were used for comparative evaluation; 13 cases were outside the approved proton range of the TPS for using the equi-spaced angle. The errors occurred in the right posterior oblique (RPO) field of 120°.

For the evaluation data, the mean CIs for PGTV were 1.04, 0.99, and 1.17 for BAODS-Net, equi-spaced angle, and planner, respectively. The mean (standard deviation) V_27Gy_ values for TLV were 10.3% (5.4), 10.6% (5.3), and 10.3% (5.3), respectively. The mean (standard deviation) V_30Gy_ values for TLV were 9.7% (5.1), 9.8% (5.0), and 9.7% (5.0), respectively. Although V_27Gy_ and V_30Gy_ of Plan_Equi_ were higher than other plans, the Plan_BAO_ had no statistically significant difference with both Plan_Clinic_ (*P* = .94) and Plan_Equi_ (*P* = .11).

**Figure 5:**
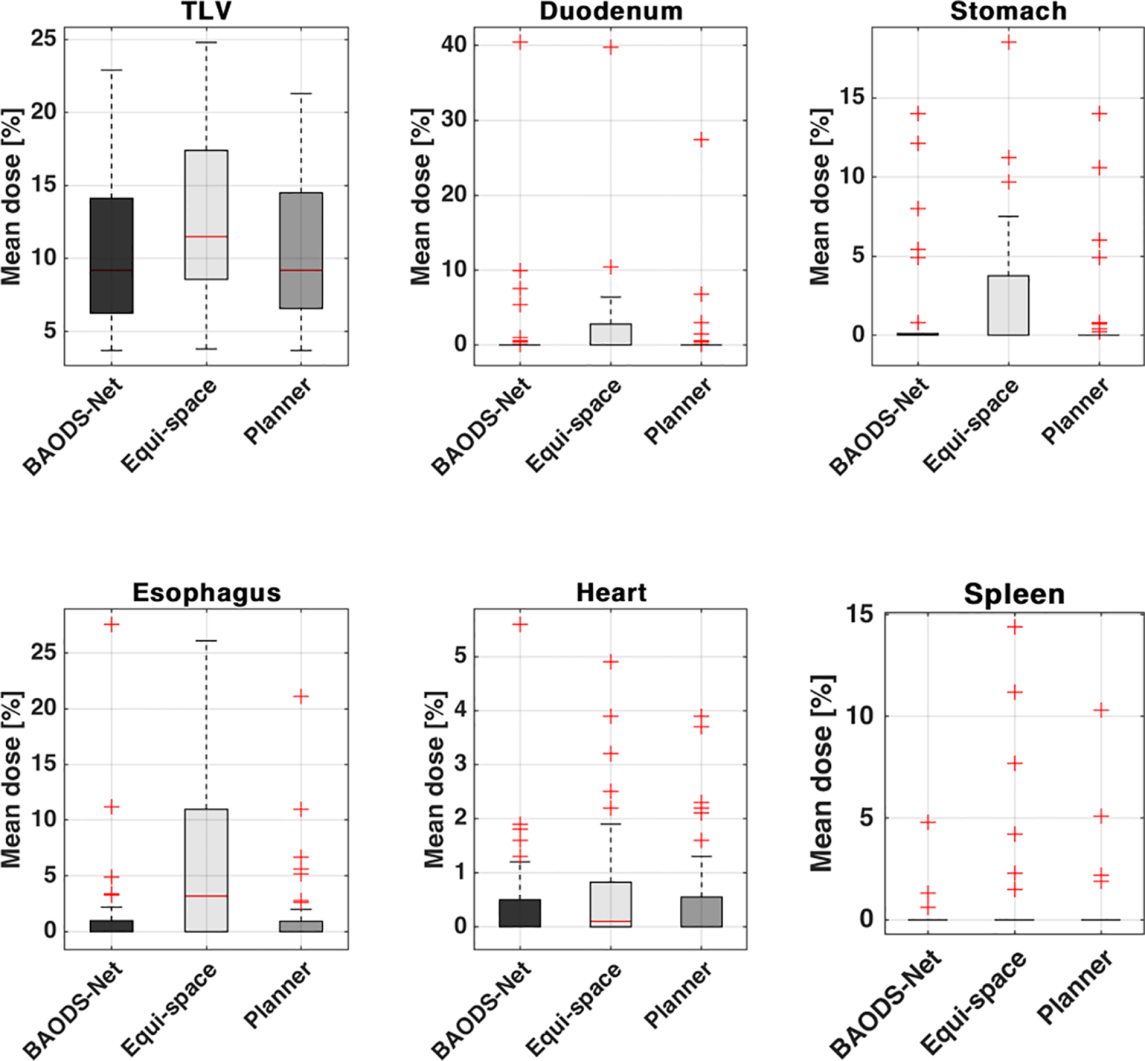
Box plot for the average of the mean dose of each structure for plans guided by BAODS-Net, equi-spaced angle, and the planner method, respectively.

For the mean dose of OARs, the results of statistical comparison between Plan_BAO_ and Plan_Clinic_ are summarized in **Table 2**. The results of statistical comparison between Plan_BAO_ and Plan_Equi_ are summarized in **Table 3**. As a statistical result, the mean dose has no significant differences between Plan_BAO_ and Plan_Clinic_ (*P* >.05), while the mean dose of Plan_Equi_ has a significant difference with Plan_BAO_ (*P* <.05). These results signified that Plan_BAO_ is superior to Plan_Equi_ and similar to Plan_Clinic_ in OARs. The mean dose is visualized for each structure in **Figure 5** as a boxplot. The central mark (red) indicates the median, and the top and bottom edges of the box indicate the 25^th^ and 75^th^ percentiles, respectively. The whiskers (-) extend to the most extreme data points, while not considering outliers (+). **Table 4** summarizes the average of the mean, minimum, maximum doses of OARs for the three planning methods. As a result, guidance using the BAODS-Net method may engender a plan with a quality similar to that created by the planner. In the case of the equi-spaced plan, the quality is relatively low compared to that of the clinical plan.

**Table 2:** Comparison of mean dose for organs-at risk-between Plan_BAO_ and Plan_Clinic_.

	Normalized Mean Dose (%)		Mean Difference (95% CI)		*p*-value
	Plan_BAO_ (*N* = 37)	Plan_Clinic_ (*N* = 37)			
Total liver volume					
Mean ± SD	10.7 ± 5.3	10.7 ± 5.2	0.051	(-0.335, 0.438)	0.7891^a^
Median (min–max)	9.2 (3.7–22.9)	9.2 (3.7–21.3)			
Duodenum					
Mean ± SD	1.8 ± 6.9	1.1 ± 4.6	0.678	(-0.115, 1.472)	0.0913^a^
Median (min–max)	0 (0–40.4)	0 (0–27.4)			
Stomach					
Mean ± SD	1.3 ± 3.3	1.0 ± 3.0	0.235	(-0.430, 0.900)	0.4778^a^
Median (min–max)	0 (0–14)	0 (0–14)			
Esophagus					
Mean ± SD	1.6 ± 4.9	1.6 ± 4.1	<0.001	(-0.551, 0.551)	1.0000^a^
Median (min–max)	0 (0–27.6)	0 (0–21.1)			
Heart					
Mean ± SD	0.5 ± 1.0	0.6 ± 1.0	-0.081	(-0.254, 0.092)	0.3473^a^
Median (min–max)	0 (0–5.6)	0 (0–3.9)			
Spleen					
Mean ± SD	0.2 ± 0.8	0.5 ± 1.9	-0.346	(-0.915, 0.223)	0.2252^a^
Median (min–max)	0 (0–4.8)	0 (0–10.3)			

a Paired samples t-test.

**Table 3:** Comparison of mean dose for organs-at-risk between Plan_BAO_ and Plan_Equi_.

	Normalized Mean Dose (%)		Mean Difference (95% CI)		*p*-value
	Plan_BAO_ (*N* = 37)	Plan_Equi_ (*N* = 37)			
Total liver volume					
Mean ± SD	10.7 ± 5.3	12.9 ± 5.8	-2.195	(-2.981, -1.409)	<.0001^a^
Median (min–max)	9.2 (3.7–22.9)	11.5 (3.8–24.8)			
Duodenum					
Mean ± SD	1.8 ± 6.9	2.3 ± 6.8	-0.565	(-1.056, -0.074)	0.0253^a^
Median (min–max)	0 (0–40.4)	0 (0–39.8)			
Stomach					
Mean ± SD	1.3 ± 3.3	2.4 ± 4.2	-1.132	(-2.069, -0.196)	0.0192^a^
Median (min–max)	0 (0–14)	0 (0–18.5)			
Esophagus					
Mean ± SD	1.6 ± 4.9	6.5 ± 8	-4.868	(-7.270, -2.465)	0.0002^a^
Median (min–max)	0 (0–27.6)	3.2 (0–26.1)			
Heart					
Mean ± SD	0.5 ± 1	0.7 ± 1.2	-0.230	(-0.409, -0.050)	0.0136^a^
Median (min–max)	0 (0–5.6)	0.1 (0–4.9)			
Spleen					
Mean ± SD	0.2 ± 0.8	1.3 ± 3.4	-1.143	(-2.109, -0.178)	0.0216^a^
Median (min–max)	0 (0–4.8)	0 (0–14.4)			

a Paired samples t-test.

**Table 4:** Average of dose–volume histogram parameters of organs-at-risk for the three planning methods.

	Minimum dose (%)	Maximum dose (%)	Mean dose (%)	Conformity index
BODY (BAODS-Net)	0.00	104.78	1.78	0.00
BODY (Equi-spaced angle)	0.00	**106.42**	**2.31**	0.00
BODY (Planner)	0.00	102.24	1.80	0.00
TLV (BAODS-Net)	0.00	104.11	10.72	0.00
TLV (Equi-spaced angle)	0.00	**106.10**	**12.92**	0.00
TLV (Planner)	0.00	104.66	10.67	0.00
PGTV (BAODS-Net)	**95.28**	104.78	101.71	1.04
PGTV (Equi-spaced angle)	93.96	**106.41**	**102.83**	0.99
PGTV (Planner)	92.53	102.62	99.32	**1.17**
Duodenum (BAODS-Net)	0.00	12.91	1.76	0.00
Duodenum (Equi-spaced angle)	0.00	**16.22**	**2.33**	0.00
Duodenum (Planner)	0.00	9.55	1.09	0.00
Stomach (BAODS-Net)	0.00	16.00	1.25	0.00
Stomach (Equi-spaced angle)	0.00	**21.11**	**2.38**	0.00
Stomach (Planner)	0.00	11.76	1.02	0.00
Esophagus (BAODS-Net)	0.00	13.94	1.62	0.00
Esophagus (Equi-spaced angle)	0.00	**23.81**	**6.49**	0.00
Esophagus (Planner)	0.00	12.36	1.62	0.00
Heart (BAODS-Net)	0.00	27.00	0.48	0.00
Heart (Equi-spaced angle)	0.00	**28.04**	**0.71**	0.00
Heart (Planner)	0.00	26.80	0.56	0.00
Spleen (BAODS-Net)	0.00	2.42	0.18	0.00
Spleen (Equi-spaced angle)	0.00	**5.47**	**1.32**	0.00
Spleen (Planner)	0.00	4.94	0.53	0.00

A higher score from the comparison of the three methods is highlighted in bold for organs-at-risk. Bold values stand for having statistical significance. TLV, total liver volume; PGTV, primary gross tumor volume.

## Discussion

The conventional procedure for creating a proton DS treatment plan is time-consuming and planner dependent. BAO can be utilized as a logical step for the development of efficient and optimal proton plans, similar to studies finding optimal fields in the static IMRT planning area. To date, there is no clinically applicable commercial software for BAO or for enabling intuitive comprehension by a planner.

In this study, we designed BAODS-Net, a new deep-learning based method of BAO for proton therapy. BAODS-Net is based on a CNN and employs the patient anatomy and HU data from the DICOM-RT structure file and AIP CT images, which are automatically extracted using the Eclipse API. The output is the predicted *S*_beam_ as angle ranking information, which could be used as *a priori* knowledge to guide the determination of the three gantry angles used for clinical practice.

According to the study results, the proposed method produced clinically acceptable and practical plans. The time required to create a proton DS treatment plan was decreased to approximately 5 min; specifically, the predicted *S*_beam_ was calculated within approximately 0.2 s through BAODS-Net.

This study provides key contributions and is distinguished from recent BAO research in several ways. This study is the first to employ the BAO method of the proton DS delivery technique, and a deep-learning based one-stop solution was developed. By leveraging this solution, a planner can refer to the predicted *S*_beam_ in the commercial TPS using Eclipse API. In contrast, conventional BAO research required multiple steps to solve the BAO problem, specifically optimizing the fluence map and then computing the dose influence matrices. However, in our research, only the patient CT anatomy and HU data were used for BAO procedures without dosimetric information from candidate beams. Similarly, Barkousaraie et al. (34) proposed a BAO method using the art column generation (CG) method and a CNN. The architecture of their deep-learning model is based on U-net (45), and the model is trained to mimic the result of the CG algorithm by using only extracted features from the patient anatomy. Although this approach also does not directly use dosimetric information for BAO, more time and additional effort are required to obtain the CG algorithm results.

The following factors may have affected the measurement accuracy. The predicted beam could not provide an optimal beam configuration because a reference *S*_beam_ was originated from Plan_Clinic_. In addition, the Plan_BAO_ was generated without manual modification/optimization procedure such as beam weight, collimator design, compensator design, *etc.* In other words, it means that the plan quality of Plan_BAO_ has the scope for improvement. In this study, we considered only coplanar proton DS plans for liver cases. However, if the search space is expanded for a non-coplanar proton DS plan, the proposed method could be applied to a non-coplanar proton DS plan for other diseases. Meanwhile, it should be noted that the field design of Plan_Equi_, specifically anterior–posterior, RPO, and left posterior oblique can be disadvantageous for liver cases. However, according to **Figure 5** and **Tables 2**–**4**, it can be confirmed that Plan_BAO_ can create a plan of similar quality to that of Plan_Clinic_ by considering the patient CT anatomy and HU data.

## Conclusion

In this paper, we validated the feasibility of using BAODS-Net for BAO of the three-port proton DS plan. BAODS-Net only used geometric information automatically extracted through the Eclipse API and could successfully predict the *S*_beam_ for the planning. The results clearly showed its potential for facilitating the three-port proton DS planning. The BAODS-Net dramatically reduced the planning time and brought us one step closer to real-time adaptive proton radiotherapy. Finally, the quality of Plan_BAO_ was statistically verified to be similar to that of Plan_Clinic_ in the mean dose of OARs (*P* >.05)

## Data Availability Statement

The datasets presented in this study can be found in online repositories. The names of the repository/repositories and accession number(s) can be found below: 10.6084/m9.figshare.14034143.

## Author Contributions

WC and HK contributed to the conception and design of the study. WC, SA, and SJ organized the database. WC, SY, and SL performed the statistical analysis. HK, SM, and TK provided guidance on methodology and the overall project. SBL, DS, YL, and JJ provided lab and technical support. WC wrote the first draft of the manuscript. All authors contributed to the article and approved the submitted version.

## Funding

This research was funded by the National Research Foundation of the Korean National Cancer Centre Fund, grant number 2110610-1.

## Conflict of Interest

The authors declare that the research was conducted in the absence of any commercial or financial relationships that could be construed as a potential conflict of interest.

## Publisher’s Note

All claims expressed in this article are solely those of the authors and do not necessarily represent those of their affiliated organizations, or those of the publisher, the editors and the reviewers. Any product that may be evaluated in this article, or claim that may be made by its manufacturer, is not guaranteed or endorsed by the publisher.

## References

[1] Taasti VT Hong L Shim JSA Deasy JO Zarepisheh M. Automating Proton Treatment Planning With Beam Angle Selection Using Bayesian Optimization. Med Phys (2020) 47(8):3286–96. doi: 10.1002/mp.14215 PMC7429260 32356335

[2] Steadham AM Liu HH Crane CH Janjan NA Rosen II. Optimization of Beam Orientations and Weights for Coplanar Conformal Beams in Treating Pancreatic Cancer. Med Dosim (1999) 24:265–71. doi: 10.1016/S0958-3947(99)00028-X 10643735

[3] Rowbottom CG Oldham M Webb S. Constrained Customization of non-Coplanar Beam Orientations in Radiotherapy of Brain Tumours. Phys Med Biol (1999) 44:383. doi: 10.1088/0031-9155/44/2/007 10070789

[4] Pugachev A Xing L. Incorporating Prior Knowledge Into Beam Orientation Optimization in IMRT. Int J Radiat Onc Biol Phys (2002) 54:1565–74. doi: 10.1016/S0360-3016(02)03917-2 12459386

[5] Morrill SM Lane RG Jacobson G Rosen II. Treatment Planning Optimization Using Constrained Simulated Annealing. Phys Med Biol (1991) 36:1341. doi: 10.1088/0031-9155/36/10/004 1745662

[6] Bortfeld T Schlegel W. Optimization of Beam Orientations in Radiation Therapy: Some Theoretical Considerations. Phys Med Biol (1993) 38:291. doi: 10.1088/0031-9155/38/2/006 8437999

[7] Lu HM Kooy HM Leber ZH Ledoux RJ. Optimized Beam Planning for Linear Accelerator-Based Stereotactic Radiosurgery. Int J Radiat Onc Biol Phys (1997) 39:1183–9. doi: 10.1016/S0360-3016(97)00344-1 9392561

[8] Pugachev A Li JG Boyer AL Hancock SL Le QT Donaldson SS. Role of Beam Orientation Optimization in Intensity-Modulated Radiation Therapy. Int J Radiat Onc Biol Phys (2001) 50:551–60. doi: 10.1016/S0360-3016(01)01502-4 11380245

[9] Pugachev A Boyer A Xing L. Beam Orientation Optimization in Intensity-Modulated Radiation Treatment Planning. Med Phys (2000) 27:1238–45. doi: 10.1118/1.599001 10902552

[10] Cho BC Roa WH Robinson D Murray B. The Development of Target-Eye-View Maps for Selection of Coplanar or Noncoplanar Beams in Conformal Radiotherapy Treatment Planning. Med Phys (1999) 26:2367–72. doi: 10.1118/1.598751 10587218

[11] Woudstra E Storchi P. Constrained Treatment Planning Using Sequential Beam Selection. Phys Med Biol (2000) 45:2133. doi: 10.1088/0031-9155/45/8/306 10958185

[12] Pugachev A Xing L. Pseudo Beam’s-Eye–View as Applied to Beam Orientation Selection in Intensity-Modulated Radiation Therapy. Int J Radiat Onc Biol Phys (2001) 51:1361–70. doi: 10.1016/S0360-3016(01)01736-9 11728698

[13] Meedt G Alber M Nüsslin F. Non-Coplanar Beam Direction Optimization for Intensity-Modulated Radiotherapy. Phys Med Biol (2003) 48:2999. doi: 10.1088/0031-9155/48/18/304 14529207

[14] Das S Cullip T Tracton G Chang S Marks L Anscher M. Beam Orientation Selection for Intensity-Modulated Radiation Therapy Based on Target Equivalent Uniform Dose Maximization. Int J Radiat Onc Biol Phys (2003) 55:215–24. doi: 10.1016/S0360-3016(02)03817-8 12504056

[15] Woudstra E Heijmen BJ. Automated Beam Angle and Weight Selection in Radiotherapy Treatment Planning Applied to Pancreas Tumors. Int J Radiat Onc Biol Phys (2003) 56:878–88. doi: 10.1016/S0360-3016(03)00266-9 12788198

[16] Woudstra E Heijmen BJ Storchi PR. Automated Selection of Beam Orientations and Segmented Intensity-Modulated Radiotherapy (Imrt) for Treatment of Oesophagus Tumors. Radiother Onc (2005) 77:254–61. doi: 10.1016/j.radonc.2005.06.028 16026873

[17] Engel K Tabbert E. Fast Simultaneous Angle, Wedge, and Beam Intensity Optimization in Inverse Radiotherapy Planning. Optimiz Eng (2005) 6:393–419. doi: 10.1007/s11081-005-2065-3

[18] Meyer J Hummel SM Cho PS Austin-Seymour MM Phillips MH. Automatic Selection of non-Coplanar Beam Directions for Three-Dimensional Conformal Radiotherapy. Br J Radiol (2005) 78:316–27. doi: 10.1259/bjr/13015047 15774592

[19] Ranganathan V Sathiya Narayanan VK Bhangle JR Gupta KK Basu S Maiya V. An Algorithm for Fast Beam Angle Selection in Intensity Modulated Radiotherapy. Med Phys (2010) 37:6443–52. doi: 10.1118/1.3517866 21302800

[20] Das SK Marks LB. Selection of Coplanar or Noncoplanar Beams Using Three-Dimensional Optimization Based on Maximum Beam Separation and Minimized Nontarget Irradiation. Int J Radiat Onc Biol Phys (1997) 38:643–5. doi: 10.1016/S0360-3016(97)89489-8 9231691

[21] Wang X Zhang X Dong L Liu H Gillin M Ahamad A. Effectiveness of Noncoplanar IMRT Planning Using a Parallelized Multiresolution Beam Angle Optimization Method for Paranasal Sinus Carcinoma. Int J Radiat Onc Biol Phys (2005) 63:594–1. doi: 10.1016/j.ijrobp.2005.06.006 16168851

[22] Oliver M Gladwish A Craig J Chen J Wong E. Incorporating Geometric Ray Tracing to Generate Initial Conditions for Intensity Modulated Arc Therapy Optimization. Med Phys (2008) 35:3137–50. doi: 10.1118/1.2937650 18697539

[23] Jia X Men C Lou Y Jiang S. Beam Orientation Optimization for Intensity Modulated Radiation Therapy Using Adaptive L2, 1–Minimization. Phys Med Biol (2011) 56:6205. doi: 10.1088/0031-9155/56/19/004 21891848

[24] Schreibmann E Xing L. Feasibility Study of Beam Orientation Class-Solutions for Prostate IMRT: Beam Orientation Class-Solutions for Prostate IMRT. Med Phys (2004) 31:2863–70. doi: 10.1118/1.1797571 15543796

[25] Bangert M Unkelbach J. Accelerated Iterative Beam Angle Selection in IMRT. Med Phys (2016) 43:1073–82. doi: 10.1118/1.4940350 26936695

[26] Ezzell GA. Genetic and Geometric Optimization of Three-Dimensional Radiation Therapy Treatment Planning. Med Phys (1996) 23:293–305. doi: 10.1118/1.597660 8815371

[27] Langer M Brown R Morrill S Lane R Lee O. A Generic Genetic Algorithm for Generating Beam Weights. Med Phys (1996) 23:965–71. doi: 10.1118/1.597858 8798167

[28] Wu X Zhu Y Dai J Wang Z. Selection and Determination of Beam Weights Based on Genetic Algorithms for Conformal Radiotherapy Treatment Planning. Phys Med Biol (2000) 45:2547. doi: 10.1088/0031-9155/45/9/308 11008955

[29] Li Y Yao J Yao D. Automatic Beam Angle Selection in IMRT Planning Using Genetic Algorithm. Phys Med Biol (2004) 49:1915. doi: 10.1088/0031-9155/49/10/007 15214533

[30] Hosseini-Ashrafi M Bagherebadian H Yahaqi E. Pre-Optimization of Radiotherapy Treatment Planning: An Artificial Neural Network Classification Aided Technique. Phys Med Biol (1999) 44:1513. doi: 10.1088/0031-9155/44/6/306 10498520

[31] Rowbottom CG Webb S Oldham M. Beam-Orientation Customization Using an Artificial Neural Network. Phys Med Biol (1999) 44:2251. doi: 10.1088/0031-9155/44/9/312 10495119

[32] Llacer J Li S Agazaryan N Promberger C Solberg TD. Non-Coplanar Automatic Beam Orientation Selection in Cranial IMRT: A Practical Methodology. Phys Med Biol (2009) 54:1337. doi: 10.1088/0031-9155/54/5/016 19204383

[33] Skrobala A Malicki J. Beam Orientation in Stereotactic Radiosurgery Using an Artificial Neural Network. Radiother Onc (2014) 111:296–300. doi: 10.1016/j.radonc.2014.03.010 24833559

[34] Sadeghnejad Barkousaraie A Ogunmolu O Jiang S Nguyen D. A Fast Deep Learning Approach for Beam Orientation Optimization for Prostate Cancer Treated With Intensity-Modulated Radiation Therapy. Med Phys (2020) 47:880–97. doi: 10.1002/mp.13986 PMC7849631 31868927

[35] Lomax A. Intensity Modulation Methods for Proton Radiotherapy. Phys Med Biol (1999) 44:185. doi: 10.1088/0031-9155/44/1/014 10071883

[36] Gu W O'Connor D Nguyen D Yu VY Ruan D Dong L. Integrated Beam Orientation and Scanning-Spot Optimization in Intensity-Modulated Proton Therapy for Brain and Unilateral Head and Neck Tumors. Med Phys (2018) 45:1338–50. doi: 10.1002/mp.12788 PMC5904040 29394454

[37] Gu W Neph R Ruan D Zou W Dong L Sheng K. Robust Beam Orientation Optimization for Intensity-Modulated Proton Therapy. Med Phys (2019) 46:3356–70. doi: 10.1002/mp.13641 PMC6692214 31169917

[38] Cao W Lim GJ Lee A Li Y Liu W Ronald Zhu X. Uncertainty Incorporated Beam Angle Optimization for IMPT Treatment Planning. Med Phys (2012) 39:5248–56. doi: 10.1118/1.4737870 PMC3422361 22894449

[39] Gu W O'Connor D Nguyen D Yu VY Ruan D Dong L. Integrated Beam Angle and Scanning Spot Optimization for Intensity Modulated Proton Therapy. Int J Radiat Onc Biol Phys (2017) 99:S107. doi: 10.1016/j.ijrobp.2017.06.254

[40] Lee N Kim TY Kang DY Choi JH Jeong JH Shin D. Development of Manual Multi-Leaf Collimator for Proton Therapy in National Cancer Center. Prog Med Phys (2015) 26(4):250–57. doi: 10.14316/pmp.2015.26.4.250

[41] Nie Y De Santis L Carratù M O’Nils M Lundgren J Sommella P. Deep Melanoma Classification With K-Fold Cross-Validation for Process Optimization. Bari, Italy: 2020 IEEE Int Sympos Med Measure Appl (MeMeA) (2020) p. 1–6.

[42] Ioffe S Szegedy C. Batch Normalization: Accelerating Deep Network Training by Reducing Internal Covariate Shift. Proc 32nd Int Conf Mach Learning PMLR (2015) 37:448–56.

[43] Xu B Wang N Chen T Li M. Empirical Evaluation of Rectified Activations in Convolutional Network. arXiv:1505.00853 (2015).

[44] Kingma DP Ba J. Adam: A Method for Stochastic Optimization. arXiv:14126980 (2014).

[45] Ronneberger O Fischer P Brox T. U-Net: Convolutional Networks for Biomedical Image Segmentation. Springer, Cham: Med Image Comput Assist Interv (2015). p. 234–41.

